# Long-term changes in the choroidal thickness in patients with unilateral central retinal vein occlusion

**DOI:** 10.1038/s41598-023-30239-1

**Published:** 2023-03-06

**Authors:** Daniel Duck-Jin Hwang, Hak Jun Lee

**Affiliations:** 1 Department of Ophthalmology, Hangil Eye Hospital, 35 Bupyeong-Daero, Bupyeong-Gu, Incheon, 21388 South Korea; 2 Department of Ophthalmology, Catholic Kwandong University College of Medicine, Incheon, South Korea

**Keywords:** Eye diseases, Retinal diseases

## Abstract

The purpose of this study was to investigate the long-term changes in the choroidal thickness in central retinal vein occlusion (CRVO) following anti-vascular endothelial growth factor (VEGF) treatment. This retrospective study included 41 eyes from 41 patients with treatment-naïve unilateral CRVO. We compared the best-corrected visual acuity (BCVA), subfoveal choroidal thickness (SFCT), and central macular thickness (CMT) of CRVO eyes with those of fellow eyes at baseline, 12 months, and 24 months. Baseline SFCT was significantly higher in CRVO eyes than in fellow eyes (*p* < 0.001); however, there was no significant difference in the SFCT between CRVO eyes and fellow eyes at 12 months and 24 months. When compared with baseline SFCT, SFCT significantly decreased at 12 months and 24 months in CRVO eyes (all *p* < 0.001). In patients with unilateral CRVO, SFCT in the CRVO eye was significantly thicker than in the fellow eye at baseline, and after 12 months and 24 months, there was no difference from the fellow eye.

## Introduction

Central retinal vein occlusion (CRVO) is a retinal vascular disease and a frequent cause of visual morbidity in older age groups, resulting from a circulatory disturbance at the trunk of the central retinal vein near the lamina cribrosa^1,2^. In addition, macular edema, intraretinal hemorrhage, and retinal ischemia can occur, resulting in significantly reduced vision^3–5^. Unlike branch retinal vein occlusion (BRVO), it is difficult to identify the obstructed site in the retina in CRVO, and it is believed that occlusion occurs inside the optic nerve; however, the exact mechanism is not known. The pathogenesis of this disease is not fully understood, and is likely multifactorial varying according to the clinical scenario.

Studies have demonstrated that atherosclerotic changes in the artery may result in narrowing of the venous lumen within the common adventitial sheath at the lamina cribrosa^6,7^. Opremcak et al. hypothesized that neurovascular compression of the central retinal artery, central retinal vein, and optic nerve head within the confined space of the scleral outlet resulting in a “compartment syndrome” occurrence in CRVO^8,9^. It has also been hypothesized that inflammation of the central retinal or peripapillary veins may be one of the causes of CRVO^3,10^. CRVO is classified into two clinical types: non-ischemic CRVO and ischemic CRVO^11^. Recently, anti-vascular endothelial growth factor (VEGF) therapy has become the gold standard treatment for macular edema associated with CRVO^12–15^.

As the choroid can be observed more precisely through enhanced depth imaging spectral-domain optical coherence tomography (EDI-OCT), many studies have been conducted to evaluate the choroidal thickness in CRVO^16–19^. Tsuiki et al. reported that the thickness of the choroid was significantly greater in the eyes with CRVO in the early stages of CRVO and decreased following intravitreal bevacizumab treatment^17^. On the other hand, Du et al. demonstrated that there was no significant difference in the choroidal thickness between CRVO eyes and normal fellow eyes^19^. As such, the results did not match the changes in the choroidal thickness in eyes with CRVO. In addition, to our knowledge, no studies have identified choroidal thickness changes for up to 2 years. Therefore, we aimed to investigate the choroidal thickness in CRVO over a long period of 2 years.

## Results

This study included 41 eyes from 41 patients with CRVO. The average age of the 41 patients with CRVO was 65.24 ± 11.87 and 17 (41.5%) were male (Table 1). Among them, 30 (73.2%) had hypertension and 19 (47.5%) had diabetes. For all periods up to 24 months, the CRVO eyes had significantly lower vision than the fellow eyes. The intraocular pressure (IOP) was not significantly different between the CRVO and fellow eyes at any time point (Table 2). The Central macular thickness (CMT) in the CRVO eyes was significantly higher than that in the fellow eyes at baseline and at 12 and 24 months (Table 3).

**Table 1:** Baseline demographics in patients with central retinal vein occlusion (N = 41, 41 eyes).

	Patients with CRVO
Patients (n)	41 (41 eyes)
Age (years)	65.24 ± 11.87
Gender	
Male	17 (41.5%)
Female	24 (58.5%)
Systemic disease	
Hypertension (%)	30 (73.2%)
Diabetes (%)	19 (47.5%)
Symptom duration (month)	0.79 ± 1.28
Baseline BCVA (logMAR)	
Affected eye	0.69 ± 0.69
Fellow eye	0.11 ± 0.15
*p*-value	< *0.001*
Baseline IOP (mmHg)	
Affected eye	15.45 ± 4.24
Fellow eye	15.80 ± 2.67
*p*-value	0.994
Baseline SFCT (μm)	
Affected eye	246.49 ± 79.69
Fellow eye	228.51 ± 76.00
*p*-value	< *0.001*
Baseline axial length (mm)	
Affected eye	23.27 ± 0.98
Fellow eye	23.34 ± 1.28
*p*-value	0.60

Values are presented as number or mean ± standard deviation, unless otherwise indicated.

*CRVO* central retinal vein occlusion, *OD* right eye, *OS* left eye, *BCVA* best-corrected visual acuity, *IOP* intraocular pressure, *SFCT* subfoveal choroidal thickness.

*p-*value derived from the independent t-test for linear values and chi-square test for categorical values.

Significant values are in italics.

**Table 2:** Best corrected visual acuity and IOP variances.

	Affected eye	Fellow eye	*p*-value
BCVA (logMAR)			
Baseline	0.69 ± 0.69	0.11 ± 0.15	< *0.001*
12th months	0.64 ± 0.72	0.10 ± 0.14	< *0.001*
24th months	0.61 ± 0.74	0.13 ± 0.20	*0.001*
IOP (mmHg)			
Baseline	15.53 ± 4.22	15.79 ± 2.63	0.676
12th months	18.00 ± 8.53	15.53 ± 4.06	0.079
24th months	17.22 ± 8.30	14.92 ± 3.33	0.082

Values are presented as mean ± standard deviation unless otherwise indicated.

*BCVA* best-corrected visual acuity, *logMAR* logarithm of the minimum angle of resolution, *IOP* intraocular pressure.

Comparison between affected and fellow eyes during each period. *P*-value derived from the paired two-tailed t-test.

Significant values are in italics.

**Table 3:** Comparison of SFCT and CMT (μm) between the eyes in patients with CRVO (N = 41, 41 eyes).

	Affected eye	Fellow eye	*P*-value^a^
SFCT (µm)			
Baseline	246.49 ± 79.69	228.51 ± 76.00	< *0.001*
12th months	224.00 ± 76.39	225.35 ± 80.71	0.777
24th months	219.71 ± 71.47	226.38 ± 82.36	0.483
* p*-value^b^			
Baseline vs. 12th M	< *0.001*	0.220	
Baseline vs. 24th M	< *0.001*	0.107	
CMT (µm)			
Baseline	617.15 ± 287.62	266.58 ± 27.29	< *0.001*
12th months	335.31 ± 175.90	267.31 ± 27.23	*0.003*
24th months	348.26 ± 155.92	268.00 ± 29.15	*0.002*
* p-*value^b^			
Baseline Vs. 12th M	< *0.001*	0.273	
Baseline Vs. 24th M	< *0.001*	0.552	

Values are presented as the mean ± standard deviation, unless otherwise indicated.

*SFCT* subfoveal choroidal thickness, *CRVO* Central retinal vein occlusion.

^a^Comparison between affected and fellow eyes in each period. *p*-value derived from the paired two-tailed t-test.

^b^Comparison between baseline and follow-up for each value. *p-*value derived from the paired two-tailed t-test.

Significant values are in italics.

### Factors associated with SFCT and SFCT decrease

In multiple linear regression analysis, with adjustment for age, gender, axial length, and injection time, only age was associated with baseline SFCT of the CRVO eyes (p = 0.017; standard regression coefficient = − 0.76). Additionally, factors that could be related to the decrease in the SFCT at 12 and 24 months compared to baseline were analyzed, but none of them showed a statistically significant association, in the CRVO eyes.

### Comparison of subfoveal choroidal thickness between CRVO eyes and fellow eyes

**Figure 1:**
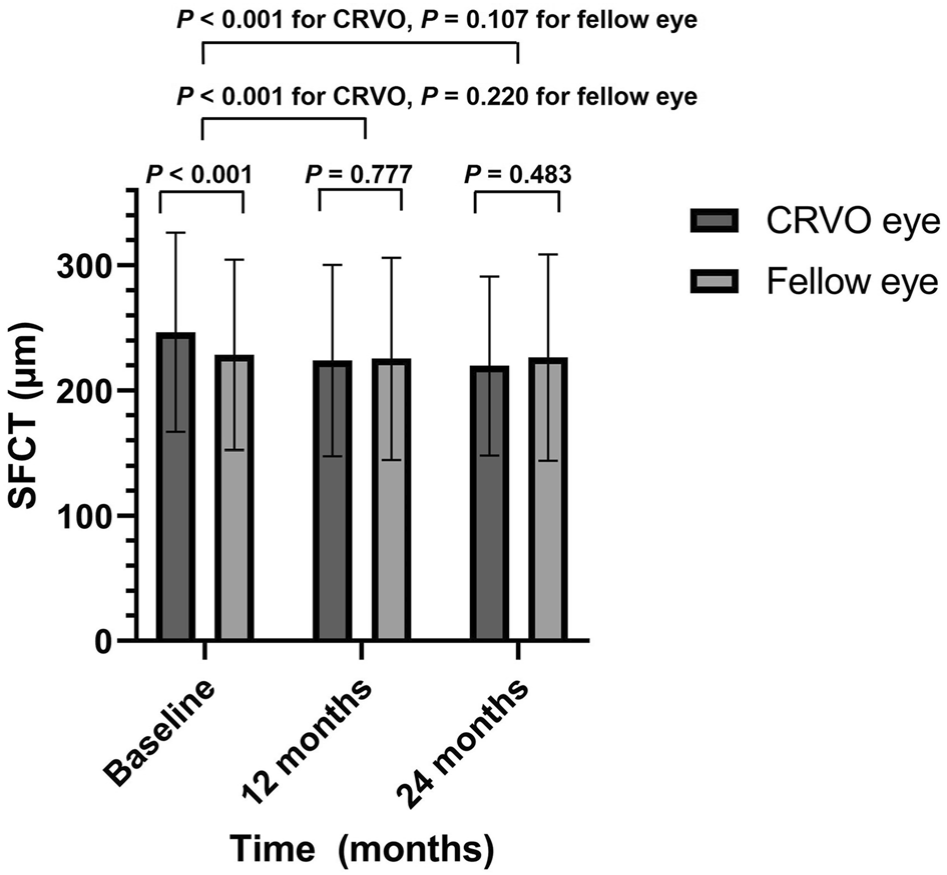
Comparison of subfoveal choroidal thickness (SFCT) (μm) between the eyes in patients with CRVO and normal fellow eyes.

In the CRVO eyes, baseline subfoveal choroidal thickness (SFCT) was significantly higher than in the fellow eyes (246.49 ± 79.69 vs. 228.51 ± 76.00, *p* < 0.001, Fig. 1). However, there was no significant difference between the two groups at 12 and 24 months (Table 3). In the CRVO eyes, SFCT decreased significantly at 12 and 24 months, compared to the baseline (*p* < 0.001 for both periods). The fellow eyes did not showed a significant decrease in the SFCT at 12 and 24 months compared to the baseline (*p* = 0.220 and *p* = 0.107, respectively; Table 3).

### Visual acuity and subfoveal choroidal thickness

Initial best-corrected visual acuity (BCVA) and the degree of vision improvement did not show a significant association with SFCT at baseline, 12 months, or 24 months. However, the worse the initial vision, the more SFCT reductions at 12 and 24 months compared to the baseline (*p* = 0.021 and *p* = 0.017, respectively).

### Intravitreal injection times and subfoveal choroidal thickness

The average number of injections in the CRVO eyes was 2.88 ± 2.41 at 12 months and 4.07 ± 3.77 at 24 months. A thicker baseline SFCT tended to have a higher total number of injections. (Spearman correlation analysis, *p* = 0.031; correlation coefficient = 0.338).

### Ischemic CRVO vs. non-ischemic CRVO

When the patients with CRVO were compared based on the classification as ischemic CRVO and non-ischemic CRVO; there was no significant difference in the SFCT and CMT in the CRVO and fellow eyes during all periods (Table 4).

**Table 4:** Comparison of SFCT and CMT between the ischemic and non-ischemic CRVO groups.

	Ischemic CRVO (n = 23)	Non-ischemic CRVO (n = 18)	*p*-value
SFCT (µm) of affected eyes			
Baseline	230.61 ± 72.77	258.91 ± 84.16	0.446
12th months	208.89 ± 70.76	235.45 ± 81.20	0.795
24th months	215.13 ± 60.60	230.67 ± 77.81	0.594
SFCT (µm) of fellow eyes			
Baseline Vs	*218.00* ± 69.43	*236.64* ± 81.36	0.685
12th months	*211.67* ± 79.05	*236.55* ± 82.15	0.459
24th months	*218.00* ± 79.64	*233.00* ± 86.02	0.784
CMT (µm) of affected eyes			
Baseline	730.06 ± 363.61	513.43 ± 168.75	0.170
12th months	352.47 ± 186.46	378.78 ± 196.38	0.227
24th months	325.13 ± 106.54	366.55 ± 183.69	0.754
CMT (µm) of fellow eyes			
Baseline	*267.72* ± 24.63	*265.64* ± 29.84	0.492
12th months	*269.06* ± 25.50	*265.95* ± 29.02	0.457
24th months	*271.06* ± 26.06	*265.42* ± 32.00	0.230

Values are presented as mean ± standard deviation unless otherwise indicated.

*BCVA* best-corrected visual acuity, *logMAR* logarithm of the minimum angle of resolution, *SFCT* subfoveal choroidal thickness, *CMT* central macular thickness.

Comparison between ischemic and non-ischemic eyes in each period. *p-*value derived from the Mann–Whitney test.

Significant values are in italics.

## Discussion

In the CRVO eyes, the initial SFCT was significantly higher than that in the fellow eyes. At 12 and 24 months, the SFCT in the CRVO was significantly reduced, and there was no difference between the CRVO and fellow eyes. Considering the CRVO eyes, the higher the initial SFCT, the greater the total number of intravitreal injections needed.

Previous studies have shown that the SFCT in eyes with CRVO was significantly thicker than that in fellow eyes, and the SFCT decreased significantly following 2 weeks of anti-VEGF treatment^20,21^. In contrast, Du et al.^19^ claimed that there was no significant change in the SFCT following VEGF treatment in long-standing CRVO eyes. In our study, the SFCT in the CRVO eyes was significantly thicker than that in the fellow eyes at baseline, and the SFCT in the CRVO eyes was significantly decreased compared to the baseline at 1 and 2 years. One of the explanations for the results that conflict with the study of Du et al.^19^ is the different phase of CRVO. Unlike this study, the study by Du et al.^19^ did not target only the recent onset of CRVO; hence, it is possible that the choroidal thickness in the CRVO eye and the fellow eye did not differ because the thickness of the choroid was already decreased in longstanding CRVO.

In CRVO, the initial CMT and CMT at 1 year and 2 years were significantly higher than those of the fellow eyes. Considering that the SFCT in the CRVO eye did not show a significant difference with the fellow eye after 1 year, the choroid remained decreased following anti-VEGF treatment; however, the macular edema seemed to persist for a longer period of time with repeated recurrences. In this study, there was no significant correlation between the CMT and SFCT at baseline, as reported by Esen et al.^22^.

A study reported that the choroidal thickness significantly decreased in CRVO eyes one month after receiving the anti-VEGF injection^17^. But in our study, the average recent injection time before the 12 and 24 months examination was − 4.86 ± 0.62 and − 9.48 ± 1.40 months, respectively. Therefore, the temporary decrease in thickness does not seem to be due to the anti-VEGF injection.

Tsuiki et al.^17^ speculated that hypoxia induced by RVO increased the VEGF, resulting in the dilatation of the blood vessels, increased blood flow, increased nitric oxide production, and vascular permeability, resulting in increased SFCT. Based on this, it was expected that the VEGF concentration in the retina and choroid would be higher in ischemic CRVO than in non-ischemic CRVO, and therefore, the SFCT might be thicker in ischemic CRVO. In this study, when comparing ischemic and non-ischemic CRVO, there was no difference in SFCT and CMT in CRVO eyes and fellow eyes. Previously, Fen et al.^21^ demonstrated that the VEGF concentration in the aqueous humor was significantly higher in ischemic CRVO than in ischemic BRVO; however, there was no significant difference in the SFCT between the two groups. Thus, if there is no significant difference in the SFCT between ischemic CRVO and non-ischemic CRVO and between ischemic CRVO and ischemic BRVO, it could be anticipated that factors other than VEGF could also influence the change in the choroidal thickness; moreover, although the SFCT increases as the VEGF concentration increases, the difference in the SFCT between the ischemic CRVO and non-ischemic CRVO groups may not appear because the choroidal thickness may not exceed a certain range in response to VEGF. Further studies are required to determine the exact mechanism.

According to prior studies, the SFCT decreased by 1.5–4.1 μm for every year as one ages^23–25^. In a central retinal artery occlusion study by Kim et al.^26^, the choroidal thickness of the contralateral eye decreased statistically significantly after 1 year (218.4 ± 111.7 μm vs. 215.4 ± 111.4 μm, p = 0.011) but the average decrease in choroidal thickness was 3 µm, which was similar to that of previous studies. In our study, the decrease in choroidal thickness of the fellow eyes was not significant after one year, and a small sample size caused by a long analysis time might have led to a selection bias. After an automated choroidal volume analysis software is developed in the near future, further studies should be performed with a large sample size to more accurately evaluate the change in choroidal thickness.

This study had several limitations. First, it was a retrospective study, and the sample size was small. The variance of SFCT is very large, but only a relatively small number of participants were included in this study. A multicenter study with a large sample size is needed in the future. Second, as this study excluded eyes that underwent PRP during follow-up in order to judge only the effect of anti-VEGF, there is a possibility that only relatively mild cases were included. Third, SFCT was manually measured at only one subfoveal point. For more objective discrimination, measuring the choroidal thickness at several sites or measuring the choroidal volume by manual segmentation of the choroidal layer^27^ would be another method. Nevertheless, this study provides important information regarding the change in the choroidal thickness in CRVO over a long period of up to 2 years.

In conclusion, in patients with unilateral CRVO, the SFCT in the CRVO eye at baseline was significantly thicker than in the fellow eye, and decreased up to 2 years, showing no difference with the fellow eye.

## Methods

### Patients

This study was a retrospective consecutive case series of patients diagnosed with treatment-naïve unilateral CRVO between January 2010 and September 2017 at the Hangil Eye Hospital. The inclusion criteria for this study were as follows: (1) symptomatic CRVO in which retinal hemorrhage and retinal edema involved the macula, (2) foveal thickness greater than 300 μm as measured by OCT at initial visits, and (3) macular edema treated with intravitreal bevacizumab. An intravitreal injection of bevacizumab was administered in the same manner as reported previously^28^. All patients were treated using a pro-re-nata regimen. The diagnosis of CRVO was based on the findings from fundus examination and fluorescein angiography. CRVO with a non-perfusion area larger than 10 disc areas on fluorescein angiography was defined as ischemic CRVO. Visual acuity improvement of 2 lines or more in the CRVO eyes following treatment was defined as a functional responder.

The exclusion criteria of the study included patients with any coexisting ocular diseases, such as age-related macular degeneration, diabetic retinopathy, and uveitis, as well as eyes that had received focal/grid laser photocoagulation, pan-retinal photocoagulation, prior intravitreal injections (e.g., intravitreal corticosteroids, intravitreal anti-VEGF agents), or prior ocular surgery (except cataract surgery). Patients were also excluded if they had refractive disorders greater than ± 3D.

Patient charts were reviewed for the following data: age, sex, medical history (hypertension and diabetes mellitus), best-corrected visual acuity (BCVA), axial length (measured with the IOL master; Carl Zeiss Meditec, Dublin, California, USA), anti-VEGF injection dates, and number of intravitreal injections. BCVA was converted to the logarithm of the minimum angle of resolution (logMAR). The BCVA, IOP, and SFCT were compared between CRVO eyes and fellow eyes at each follow-up visit.

### Imaging protocol

EDI-OCT using the Heidelberg Spectralis (Heidelberg Spectral Domain Optical Coherence Tomography; Heidelberg Engineering, Dossenheim, Germany) platform was used. SFCT was measured at the subfoveal point extending from the outer border of the pigment epithelium to the choroidal scleral boundary using the built-in caliper software. For better accuracy, horizontal and vertical macular B-scans were measured, and the choroidal thickness was determined as the average value. CMT was recorded for all patients using the automated software present in the 25-line raster scan pattern. Choroidal thickness and CMT were measured at initial presentation and at 12 and 24 months of follow-up for the CRVO and fellow eyes.

### Ethics statement

This study was conducted in accordance with the principles of the Declaration of Helsinki. The Institutional Review Board (IRB) of Hangil Eye Hospital approved this study and waived the requirement for informed consent from the study participants due to the retrospective nature of the study.

### Statistical analysis

The IBM SPSS Statistics 25.0 software for Windows (SPSS, Chicago, Illinois, USA) was used for data analysis. The SFCT was measured in the CRVO and fellow eyes, and compared using a paired t-test. The analysis of factors related to visual acuity improvement was performed using Spearman’s correlation analysis. Comparisons between ischemic and non-ischemic CRVO were performed using Wilcoxon signed-rank tests. Multiple linear regression analysis was subsequently performed using the forward selection technique to identify explanatory variables with a statistically significant contribution to baseline SFCT. Differences were considered statistically significant at *p*-values less than 0.05.

## Data Availability

The data are not available for public access because of patient privacy concerns, but are available from the corresponding author on reasonable request.
